# Research trends and hot spots in global nanotechnology applications in liver cancer: a bibliometric and visual analysis (2000-2022)

**DOI:** 10.3389/fonc.2023.1192597

**Published:** 2023-07-28

**Authors:** Xi Jin, Jingwei Zhao, Hongshuang Li, Mengting Zheng, Jiao Shao, Zhanguo Chen

**Affiliations:** Department of Clinical Laboratory, The Second Affiliated Hospital and Yuying Children’s Hospital of Wenzhou Medical University, Wenzhou, Zhejiang, China

**Keywords:** bibliometric, liver cancer, nanotechnology, visualization, hepatocellular carcinoma

## Abstract

**Background:**

Liver cancer (LC) is one of the most common malignancies. Currently, nanotechnology has made great progress in LC research, and many studies on LC nanotechnology have been published. This study aims to discuss the current status, hot spots, and research trends in this field through bibliometric analysis.

**Methods:**

The Web of Science Core Collection (WoSCC) database was searched for papers related to hepatocellular carcinoma (HCC) included from January 2000 to November 2022, and its research hotspots and trends were visualized and analyzed with the help of VOSviewer. In addition, a search was conducted to find LC papers related to nanotechnology. Then we used the visual analysis software VOSviewer and CiteSpace to evaluate the contributions of countries/regions, authors, and journals related to the topic and analyze keywords to understand the research priorities and hot spots in the field as well as the development direction.

**Results:**

There are 1908 papers in the highly cited literature on LC, and its research hotspots are pathogenesis, risk factors, and survival rate. The literature on the application of nanotechnology in LC had 921 papers. Among them, China (n=560, 60.8%) and the United States (n=170, 18.5%) were the countries with the highest number of published papers. Wang Yan (n=11) and Llovet JM (n=131) were the first authors and co-cited authors, respectively. The International Journal of Nanomedicine was the most prolific academic journal (n=41). In addition to “hepatocellular carcinoma” and “nanoparticles”, the most frequent keyword was “drug delivery”. In recent years, “metastasis” and “diagnosis” appeared in the keyword bursts. This indicates that the application of nanoparticles in the early diagnosis and drug delivery of LC (including liver metastasis) has a good prospect.

**Conclusion:**

Nanotechnology has received more and more attention in the medical field in recent years. As nanoparticles are easily localized in organelles and cells, they can increase drug permeability in tumor tissues, improve drug delivery efficiency and reduce drug toxicity. Our research results were the first scientific evaluation of the application of nanotechnology in LC, providing scholars with research hotspots and development trends.

## Introduction

One of the top causes of cancer-related fatalities globally is liver cancer (LC). About 90% of primary LC are hepatocellular carcinoma (HCC) (1–3). Primary LC is associated with viral hepatitis (hepatitis B virus (HBV) and hepatitis C virus (HCV)), metabolic alterations (alcoholic steatohepatitis (ASH), non-alcoholic steatohepatitis (NASH)) (4), chronic exposure to aflatoxin, algal toxin-contaminated water, diabetes, parasitic infections, and genetic factors (5). According to one study, there were about 840,000 confirmed cases of LC as well as 780,000 new fatalities in 2018, making it the fourth most common cancer-related cause of death globally (1). Generally, it is ignored in the early stage because of asymptomatic or atypical symptoms. When the patient presents with obvious discomforts, such as fatigue, loss of appetite, liver pain, etc., the disease is often advanced to the middle and late stages, at which time traditional treatment including surgical excision and liver transplantation has little effect. In addition, conventional chemotherapeutic drugs are not specific to tumor tissues (6, 7), and long-term chemotherapy can lead to drug toxicity and multiple drug resistance (6, 8). Despite significant advances in the pathogenesis and molecular characterization of LC in recent years, treatment options that improve patient survival and quality of life significantly are still scarce (9). Therefore, it is particularly important to find appropriate techniques to improve the early screening, accurate diagnosis, optimal efficacy, recurrence, and metastasis of LC.

Nanotechnology is now used in a variety of medical tests and screenings, such as gold nanoparticles for home pregnancy testing (10). In cancer diagnosis, nanoparticles are used to capture cancer biomarkers such as cancer-associated proteins, circulating tumor DNA, circulating tumor cells, and exosomes (11). The ratio of surface area to volume of nanoparticles is larger than that of bulk materials. Therefore, the surface of the nanoparticle can be densely covered by other parts such as antibodies, small molecules, and peptides, which can bind and recognize specific cancer molecules. By providing various binding ligands to cancer cells, multivalent effects can be achieved, thereby improving the specificity and sensitivity of detection (12). Compared to conventional therapies (e.g., chemotherapy and radiotherapy), nanoparticle drug delivery systems can avoid the body’s natural barriers, prevent early degradation or metabolism of drugs, and deliver drug molecules to their intended destinations with precision (13–15), enabling cell-specific targeting and thus effectively avoiding high doses of drugs, systemic toxicity, and normal cell damage, which greatly reduces cancer mortality (16). In addition, nanocarriers are easy to synthesize, cost-effective, and easy to customize for applications. Many studies have extensively used nanotechnology to treat various cancers, including LC. To date, the US Food and Drug Administration (FDA) has approved and marketed a handful of targeted nanomedicines, including the targeted drug sorafenib. However, there are still bottlenecks in clinical applications, such as the use of nanotechnology to improve the efficacy of nanodelivery systems such as sorafenib, and the precise diagnosis of LC, including liver metastases. Meanwhile, due to the lack of a large number of publications and quantitative analysis of all literature in this field, it is impossible to grasp the latest research trends and hot spots.

Bibliometrics is an integrated body of knowledge that integrates mathematics, statistics, and literature, emphasizing quantification. It can help researchers in different fields build knowledge maps, assess topic trends, and identify hotspots in research areas. CiteSpace and VOSviewer are the most commonly used tools to analyze academic literature visually (17). Researchers can get helpful information and research trends by combining quantitative and statistical analyses in the field (18). Currently, many articles are studying the combination of LC and nanotechnology; however, there has been no bibliometric analysis of the application of nanotechnology in LC. The purpose of this study is to explore the research process and current status of nanotechnology in LC over the past two decades using various methods and perspectives of bibliometrics, which helps scholars to understand more intuitively the research hotspots and future research directions in this field.

## Materials and methods

### Data collection

**Figure 1:**
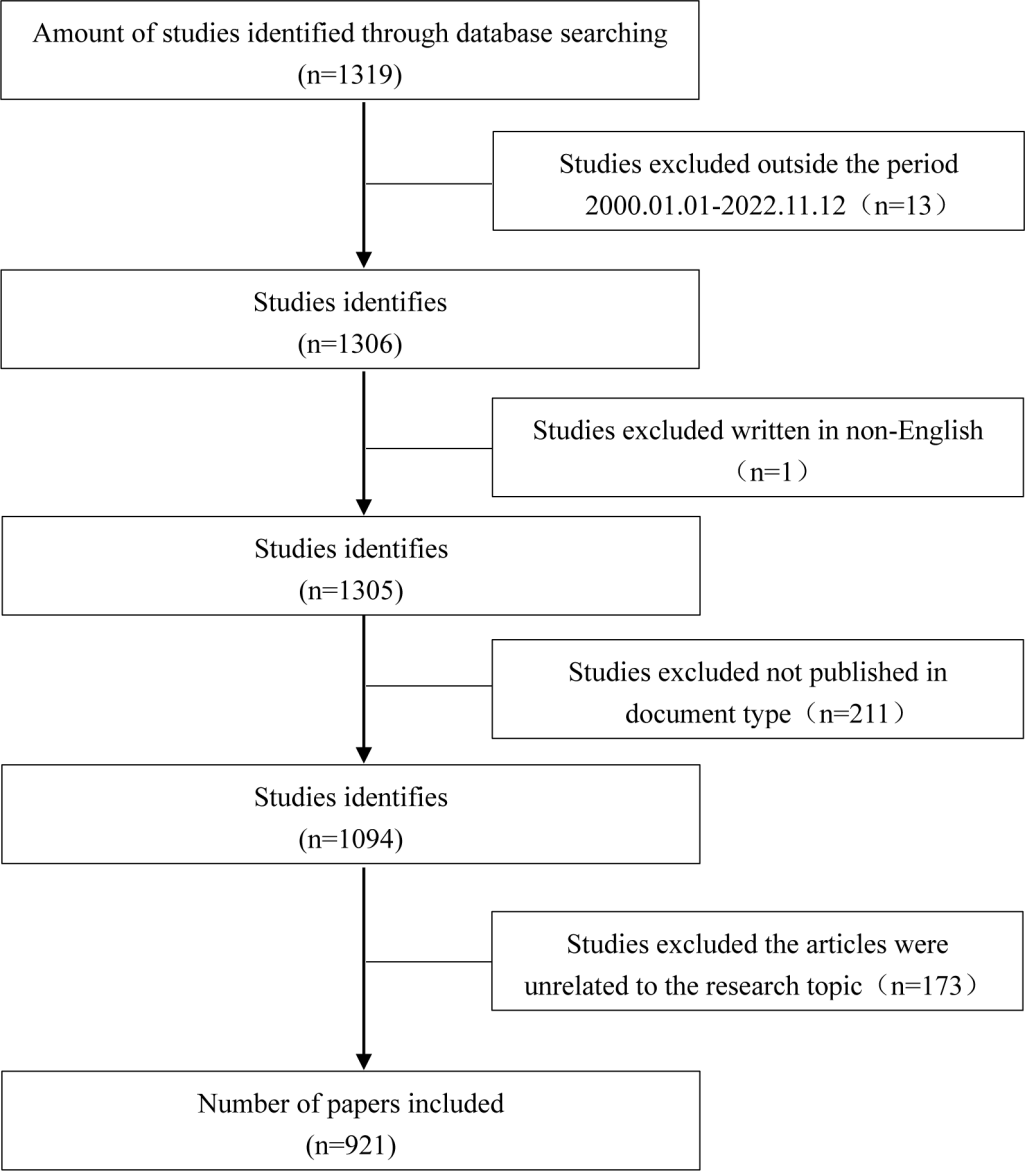
Flow chart of literature selection.

We searched the WoSCC database for papers related to nanotechnology in LC, with topics limited to (TS=(“nanotechnology” or “nanomedicine” or “nanoparticles” or “drug carriers”) and TS=(“liver cancer” or “hepatocellular carcinoma” or “liver cancer” or “liver tumor”)), with 1315 articles; the limited period was January 1, 2000, to November 12, 2022 (excluding 13 articles); language selected was English only (1 article excluded), and article type was restricted to articles (221 articles excluded). To assure the quality of the search, two independent reviewers evaluated the complete studies, discussed them when there was disagreement, and excluded 173 papers that were not relevant to the topic, the 921 papers that met the criteria were exported in the form of “complete records and citations” for subsequent data analysis. An overview of the study selection process can be seen in **Figure 1**.

### Data analysis

We mainly used Microsoft Office Excel 2016, VOSviewer 1.6.18, and CiteSpace 6.1.6 for data management. Microsoft Office Excel 2016 was used to construct a trend graph to show the number of annual publications, with function fitting based on the line chart. The percentage of the variance in the dependent variable is expressed by the coefficient of determination (R^2^). The closer the R^2^ value is to 1, the better the fitting effect will be. The closer it is to zero, the worse the fitting degree will be.

VOSviewer is a JAVA-based freeware developed in 2009 by Van Eck and Waltman at Leiden University, The Netherlands, and can be downloaded quickly and easily as an installer from this link https://www.vosviewer.com/. VOSviewer (19) is a powerful tool for mapping scientific knowledge in database formats such as Web of Science, Scopus, Dimensions, and Pubmed. It is widely used for author collaboration networks, institutional collaboration networks, and literature co-citation analysis. The software focuses on the visualization of scientific knowledge and is primarily used for textual analysis of data. In this study, VOSviewer was used to analyze network graphs about countries/regions, authors, journals, and keywords. In addition, VOSviewer provides data on countries/regions and institutions, authors and co-cited authors, journals and co-cited journals, co-cited references, and keywords. The size of the nodes on the visualization map represents the frequency of occurrence; the higher the frequency, the larger the node. In a cooperative network, the thicker the connection line, the higher the frequency of cooperation. In the cluster density map, clusters of the same color indicate close cooperation.

CiteSpace is a free Java application developed by Professor Chaomei Chen of Radissonblue University and available for free download at http://cluster.cis. It can extract authors, institutions, countries, journals, references, keywords, and other information from the literature, and then reconstruct according to the type and intensity between them to form a network structure. Nodes of the network represent units of information in the literature, and lines represent connections between nodes (co-occurrence). Finally, through measurement, statistical analysis (clustering, outburst word detection, etc.), and visualization of node, wire, and network structure, knowledge structure patterns and research trends in specific disciplines and domains are discovered (20, 21). Betweenness centrality is an important parameter of Citespace. In general, nodes with centrality ≥ 0.1 are considered more important and are also marked with a purple circle in Citespace. It is primarily a measure of the value of nodes that play a bridging role in the overall network structure (22). In this analysis, CiteSpace software was used to map the network structure of countries and institutions, the dual-map overlay of journals, the co-cited network and timeline view of journals, the cited outbreaks of the top 20 articles as well as the top 20 keywords. The parameters were set as follows: Time span:2000-2022 (section length = 1), selection criteria:g-index (k=25), link retention factor (LRF) = 3.0, year of review (LBY) = 5, top N (e) = 1.0.

## Results

### Research rends and hot spots in LC

**Figure 2:**
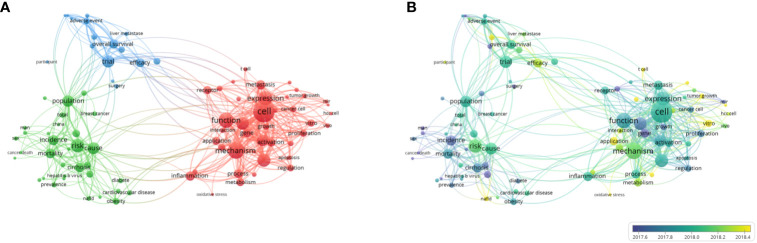
Research trends and hot spots in LC. **(A)** Network Visualization: In the network view, colors represent categories, and the same color is a keyword for a clustering category (representing a research direction). **(B)** Country distribution: The color represents the time, which is the average time this keyword appears. The default is blue and yellow, with blue appearances preceding and yellow appearances updated.

According to the search terms, a total of 46,797 articles on LC research published between January 2000 and November 2022 were retrieved from the WoSCC database. As the large number affected the operation of VOSviewer, we selected 1908 highly cited articles for analysis. The VOSviewer cluster view shows that there are three clusters of research hotspots, namely, pathogenesis (red), LC risk factors, incidence, and pathogenicity, etc. (green), and treatment effect and survival rate of LC (blue) (**Figure 2A**). In the labeled view, according to the timeline, the research trends and hot spots after April 2018 include non-alcoholic hepatitis (NAFLD), Vitro, tumor growth, etc. (**Figure 2B**).

### Distribution of LCN articles by Publication Years

According to the search terms, a total of 921 articles on nanotechnology research on LC published between January 2000 and November 2022 were retrieved from the WoSCC database. As illustrated in **Supplemental Figure 1**, the results indicate that there were few reports published on LC in nanotechnology research until 2008. From the year 2009 to 2018, the relevant publications presented a slight upward trend, peaking in 2021. According to a mathematical polynomial function fit, it can be seen that the degree of fitting is good, R^2^ = 0.949.

### Countries/regions analysis in LCN articles

**Figure 3:**
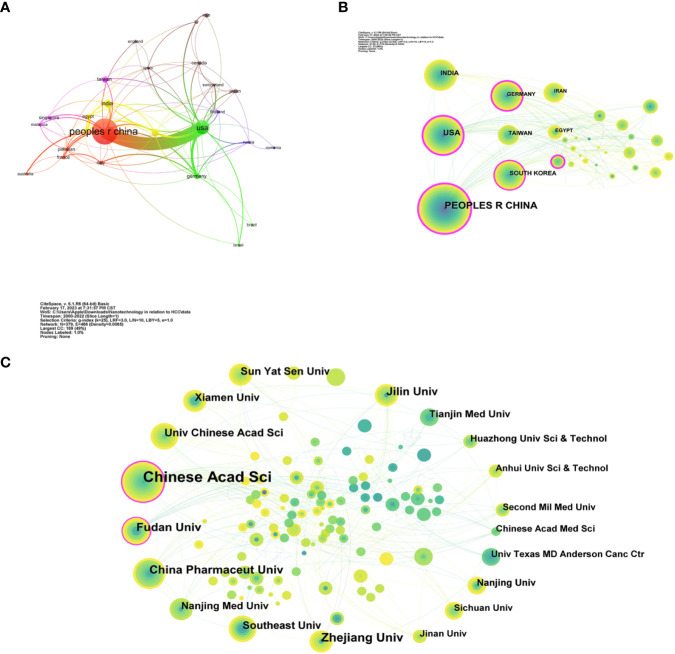
Analysis of countries/regions involved in LC nanotechnology. **(A)** National network map. **(B)** Country distribution. **(C)** Institutional distribution.

The 921 papers on LC nanotechnology published between January 2000 and November 2022 were extracted from the WoSCC database, coming from 1,144 institutions in 55 different countries/regions. In order of the author countries of the published journals, the top three were China (n=560, or 60.8%), the United States (n=170, or 18.5%), and India (55, or 6.0%) (**Table 1**). While more than half of all publications came from China, which may be explained by the high prevalence of LC in China (23). In addition, among these countries/regions, the mean citation rate and centrality were higher in the United States (41.19/54%), South Korea (33.29/0.18%), Italy (30.65/0.22%), Germany (29.41/0.38%), and China (24.75/0.38%). In addition, based on VOSviewer software, we observe strong cooperation between nations and regions, particularly between China and the United States (**Figure 3A**). It is clear that the two nations are the main exporters of scientific knowledge in this area, and they have a considerable impact on its development.

**Table 1:** Top ten countries/regions and institutions for related publications.

Rank	Countries/regions	Publications	Citations	Average Citations	Centrality	Rank	Institutions	Publications	Citations	Average Citations	Centrality
1	People’s Republic of China	560	13858	24.75	0.38	1	Chinese Acad Sci	54	2416	44.74	0.23
2	United States	170	7002	41.19	0.54	2	Zhejiang Univ	26	678	26.08	0.09
3	India	55	1187	21.58	0.04	3	Fudan Univ	24	603	25.13	0.11
4	South Korea	51	1698	33.29	0.18	4	Jilin Univ	20	955	47.75	0.08
5	Germany	32	941	29.41	0.38	5	China Pharmaceut Univ	20	563	28.15	0.08
6	Egypt	24	300	12.50	0.03	6	Sun Yat Sen Univ	20	523	26.15	0.06
7	Iran	21	516	24.57	0.00	7	Southeast Univ	20	709	35.45	0.03
8	Saudi Arabia	18	138	7.67	0.14	8	Univ Chinese Acad Sci	16	341	21.31	0.09
9	Italy	17	521	30.65	0.22	9	Xiamen Univ	16	461	28.81	0.03
10	Turkey	16	203	12.69	0.08	10	Nanjing Med Univ	15	419	27.93	0.01

A visual map of the author’s country/region or institution was generated through CiteSpace (**Figures 3B, C**). The size of the circle represents how many publications a country, region, or institution produces, and the line connecting the circles illustrates the cooperation between them. The purple ring denotes a node of high centrality. And the thicker it is, the higher the value of intermediary centrality. According to **Table 1**, the United States (centrality = 0.54) ranks first in the list of the top ten countries/regions in terms of total centrality, implying that it plays a key role as a bridge in the global network of country/region cooperation. In the future, close academic exchanges between countries/regions will contribute to the development of this research field.

### Author and co-cited author analysis in LCN articles

**Figure 4:**
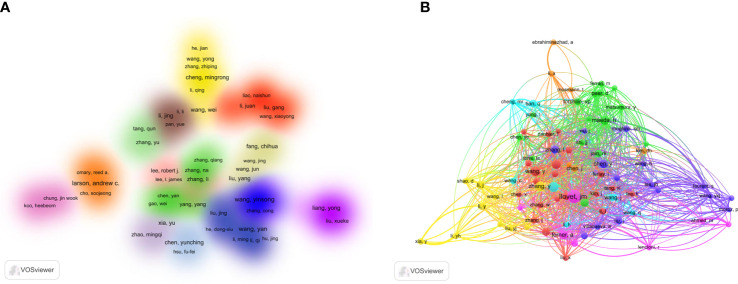
Analysis of LC nanotechnology authors and co-cited authors. **(A)** Author co-authorship analysis of VOSviewer density visualization. **(B)** Analysis of co-cited authors for VOSviewer network visualization.

From January 2000 to November 2022, 5,756 researchers were involved in linked studies on nanotechnology and LC. The co-authorship analysis of the author is depicted in **Figure 4A** using cluster density mapping. There were 238 authors total (more than 3 papers), Wang Yan ranked first, followed by Wang Yinsong, which demonstrates their influence in the field (**Table 2**).

**Table 2:** Top ten authors and co-cited authors contained research on nanotechnology to LC.

Rank	Author	Count	Rank	Co-cited Author	Count
1	Wang, Yan	11	1	Llovet, Jm	131
2	Wang, Yinsong	9	2	Bruix, J	72
3	Zhang, Ning	9	3	Wang, Y	69
4	Larson, Andrew c.	9	4	Maeda, H	67
5	Cheng, Mingrong	8	5	El-serag, Hb	62
6	Tang, Xiaolong	8	6	Zhang, Y	62
7	Liang, Yong	8	7	Forner, A	57
8	Chen, Yunching	8	8	Chen, Y	51
9	Liu, Yang	8	9	Liu, Y	48
10	Liu, Ying	8	10	Zhang, L	47

In addition, the VOSviewer density visualization graph shows that authors with close collaborations are assigned to a cluster of the same color, a total of 15 clusters shown in **Figure 4A**. Among them, Chinese author clusters make up the majority of them.

The term “co-cited authors” refers to two (or more) authors who are cited simultaneously in the same work, and who are subsequently credited with having a co-cited relationship (24). In the network, every node represents an author, while every line represents a collaboration between two authors. If the connection line is thicker, it indicates a closer partnership (**Figure 4B**). After setting the minimum number of citations to 20, the WoSCC database was used to obtain the list of co-cited authors. Among them, Llovet JM (n=131) and Bruix J (n=72) ranked at the top (**Table 2**).

### Journal and co-cited academic journals in LCN

Articles on LC nanotechnology have appeared in 335 different publications. The most published journal was the *International Journal of Nanomedicine* (n=41, IF2022 = 8.00), closely followed by *Biomaterials* (n=33, IF2022 = 13.9993). Nanomedicine and biomaterials research will remain a priority in science both today and in the future. In addition, the journal impact factor (IF) is a quantitative indicator of a journal’s impact and an important indicator for academic departments to assess academic productivity. *Acs Nano* (n=13, IF2022 = 17.100) had the top impact factor, followed closely by *Biomaterials* (n=33, IF2022 = 3.9993) (**Table 3**). All of this suggests that these journals are particularly keen on research regarding the application of LC nanotechnology. Among the co-citations, the journal with the most citations was *Biomaterials* (484) followed by Journal of *Controlled Editions* (419) and *Acs Nano* (339) (**Table 4**).

**Table 3:** Top ten academic journals about nanotechnology to LC.

Rank	Journal	Publications	Citations	Country	IF (2022)#	JCR
1	International Journal of Nanomedicine	41	1273	New Zealand	8.000	Q1
2	Biomaterials	33	2656	Netherlands	13.9993	Q1
3	Nanoscale	23	856	England	6.700	Q1
4	Journal of Biomedical Nanotechnology	21	381	United States	2.8997	Q3
5	Acs Applied Materials & Interfaces	19	444	United States	9.4998	Q1
6	Rsc Advances	16	283	England	3.9003	Q2
7	Nanomedicine-Nanotechnology Biology and Medicine	14	518	Netherlands	5.3999	Q2
8	Journal of Controlled Release	13	833	Netherlands	10.7997	Q1
9	Drug Delivery	13	275	United States	6.000	Q1
10	Acs Nano	13	1117	United States	17.100	Q1

#The impact factors (IF) of journals were obtained from the 2022 Web of Science Journal Citation Reports (JCR).

**Table 4:** Top ten Co-Cited academic journals about nanotechnology to LC.

Rank	Journal	Count	Centrality	Country	IF (2022)#	JCR
1	Biomaterials	484	0.09	Netherlands	14.000	Q1
2	Journal of Controlled Release	419	0.02	Netherlands	10.800	Q1
3	Acs Nano	339	0.03	United States	17.100	Q1
4	Advanced Drug Delivery Reviews	298	0.01	Netherlands	16.100	Q1
5	International Journal of Nanomedicine	296	0.01	New Zealand	8.000	Q1
6	Cancer Research	289	0.04	United States	11.200	Q2
7	International Journal of Pharmaceutics	263	0.02	Netherlands	5.800	Q1
8	Proceedings of the National Academy of Sciences of the United States of America	255	0.03	United States	11.100	Q1
9	Journal of the American Chemical Society	221	0.02	United States	15.000	Q1
10	Hepatology	219	0.03	United States	13.500	Q1

#The impact factors (IF) of journals were obtained from the 2022 Web of Science Journal Citation Reports (JCR).

**Figure 5:**
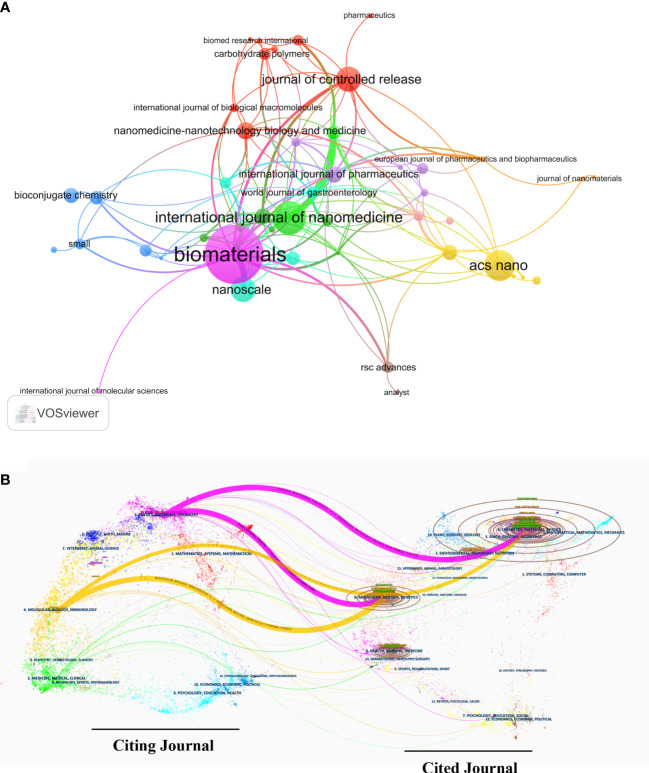
Analysis of LC nanotechnology journals and co-cited academic journals. **(A)** Network diagram of academic journals in VOSviewer. **(B)** The dual-map overlay of Citespace journals.

**Figure 5A** demonstrates the visualization map of co-citation relationships of different journals. In addition, a dual-map overlay of publications was established to show the top subject distribution of scholarly journals (25) (**Figure 5B**). Where the left side is the citing journal and the right side is the cited journal. Four distinct colored paths indicate the citation pathways in this map, with the thicker lines representing the main pathways. According to two purple citation routes, articles published in the journals Chemistry/Materials/Physics and Molecular/Biology/Genetics are commonly cited studies from journals of Physics/Materials/Chemistry. Two yellow paths showed that both Molecular/Biology/Genetics journals and Chemistry/Materials/Physics journals are commonly cited works from the Molecular/Biology/Immunology journals.

### Co-cite reference analysis in LCN

In addition to presenting the foundational works that are essential to the development of a discipline, the co-cited sources demonstrate the sources and changing trends in research in the field (26). To allow researchers to have an overview of the application of nanotechnology in LC and the structural characteristics of its development, we used VOSviewer software to screen the 10 articles with the highest total citations by title, co-author, country, journal, etc. As shown in **Table 5**, the co-cited literatures were mainly from well-known journals, and oncology-related fields accounted for most of the main fields. Among them, Bray F et al. (2018) published (1) “*Global cancer statistics 2018: incidence and mortality estimates for 36 cancers in 185 countries worldwide*” was the most co-cited (n=44) and had the highest impact factor (IF2022 = 254.700). In addition to this, there were four different countries in the top 10 references, including France, Spain, the United Nations, and Japan.

**Table 5:** Top ten Co-Cited References about nanotechnology to LC.

Rank	Title	Year	Journal	Citations	Country	IF (2022)#	JCR
1	Global cancer statistics 2018: GLOBOCAN estimates of incidence and mortality worldwide for 36 cancers in 185 countries	2018	CA-A Cancer Journal for Clinicians	44	France	254.700	Q1
2	Sorafenib in advanced liver carcinoma	2008	New England Journal of Medicine	43	Spain	158.500	Q1
3	Nanocarriers as an emerging platform for cancer therapy	2007	Nature Nanotechnology	33	United States	38.300	Q1
4	liver carcinoma	2012	Lancet	31	Spain	168.900	Q1
5	Tumor vascular permeability and the EPR effect in macromolecular therapeutics: a review	2000	Journal of Controlled Release	29	Japan	10.800	Q1
6	Global Cancer Statistics	2011	CA-A Cancer Journal for Clinicians	28	United States	254.700	Q1
7	A new concept for macromolecular therapeutics in cancer chemotherapy: mechanism of tumoritropic accumulation of proteins and the antitumor agent smancs.	1986	Cancer Research	24	Japan	11.200	Q1
8	Nanoparticle therapeutics: an emerging treatment modality for cancer	2008	Nature Reviews Drug Discovery	23	United States	120.100	Q1
9	Rapid colorimetric assay for cellular growth and survival: application to proliferation and cytotoxicity assays.	1983	Journal of Immunological Methods	23	United States	2.200	Q3
10	Cancer nanotechnology: Opportunities and challenges	2005	Nature Reviews Cancer	22	United States	78.500	Q1

#The impact factors (IF) of journals were obtained from the 2022 Web of Science Journal Citation Reports (JCR).

**Figure 6:**
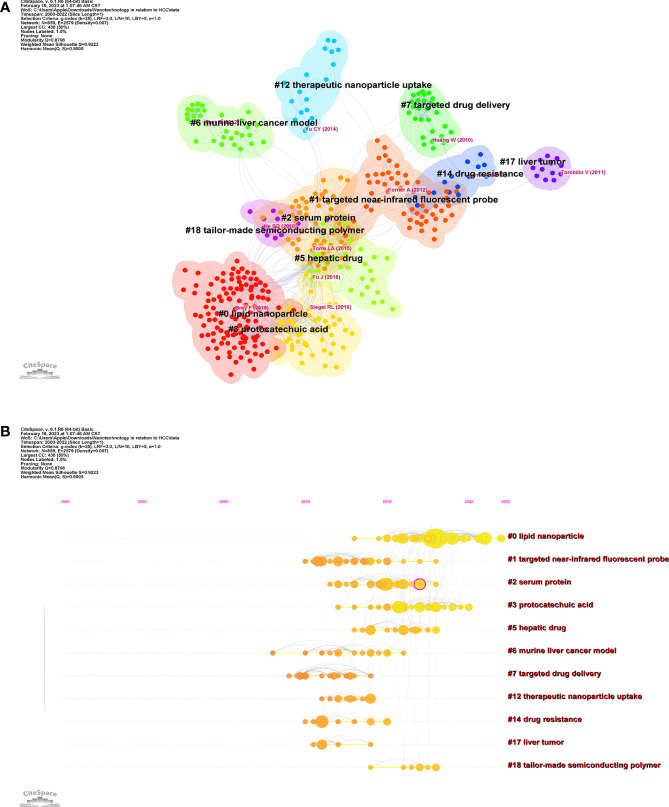
Citespace analysis of co-cited literature on nanotechnology for LC. **(A)** Co-citation network. **(B)** Timeline graph of co-cited clusters of the journal literature.

CiteSpace divides co-citation networks into 11 clusters by grouping references based on their tight or weak links (**Figure 6A**). CiteSpace provides two metrics, the modularity (Q score) and the silhouette score (S score). The range of the Q score was typically [0, 1], and empirical data showing Q > 0.3 indicated the existence of a real community structure. The S score represented the value of the average cluster profile. The clustering was reasonable if S > 0.5, while the clustering was valid and persuasive if S > 0.7 (27). In this work, the Q and S scores of this study are 0.8796 and 0.9223, respectively. As can be observed, the Q score exceeded 0.3, and the S score exceeded 0.7, demonstrating the importance of the clusters’ modular structure and the validity of the clustering effect. **Table 6** shows that the lowest S score is 0.886(#0) and the highest S score is 0.99(#17), indicating that clustering has high homogeneity.

**Table 6:** Major clusters of co-cited references of nanotechnology to LC.

Cluster-ID	Size	Silhouette	Mean Year	Top Terms
#0	125	0.913	2018	lipid nanoparticle
#1	54	0.886	2012	targeted near-infrared fluorescent probe
#2	53	0.917	2015	serum protein
#3	53	0.896	2017	protocatechuic acid
#5	34	0.891	2015	hepatic drug
#6	32	0.98	2012	murine liver cancer model
#7	28	0.94	2011	targeted drug delivery
#12	19	0.983	2012	therapeutic nanoparticle uptake
#14	14	0.969	2012	drug resistance
#17	12	0.995	2011	liver tumor
#18	12	0.99	2017	tailor-made semiconducting polymer

**Figure 7:**
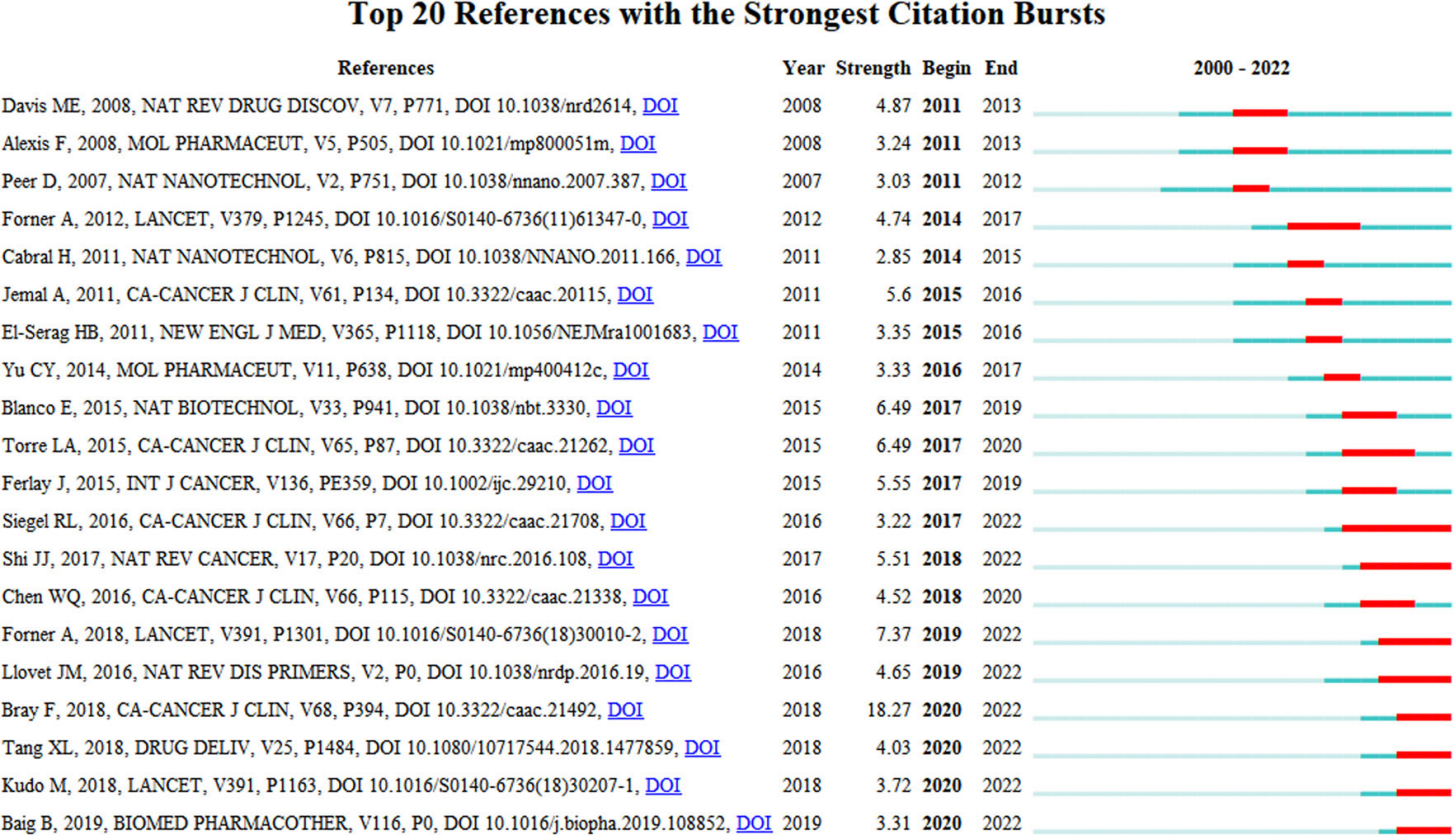
The top 20 references with the strongest citation explosion for LC nanotechnology.

Time-slicing and clustering techniques are combined in the timeline view, a way of data display. The clustering labels, which are arranged according to whether the clustering happened in the early or late periods, can show not only how study subjects are distributed across the field but also how they change with time. **Figure 6B** shows that in the timeline view, a node further to the left indicates an older reference, while a node further to the right indicates a more recent reference. The same row indicates the set to which all cluster citations are assigned, with the cluster label at the far right of the row. CiteSpace can be used to assess the citation rate of a reference document. A high number of citations over time and a well-known publication’s appearance in the area are both signs of a citation burst. We found relatively concentrated clusters in the temporal distribution mainly appearing between 2008-2022, with the early appearance being the “#6 murine liver cancer model” and more recently the “#0 lipid nanoparticles”, indicating that the #0 cluster is the most popular research direction for nanotechnology applications in LC in recent years (**Figure 6B**). Amongst the top 20 references with the most citation bursts, “*Bray F,2018, CA-CANCER J CLIN, V68, P394, DOI 10.3322/caac.21492” (2020-2022, strength18.27)* and “*Tang XL,2018, DRUG DELIV, V25, P1484, DOI 10.1080/10717544.2018.1477859*” (2020-2022, strength 4.03) was the recent emergence of high-citation references (**Figure 7**).

### Keyword visualization analysis in LCN

**Figure 8:**
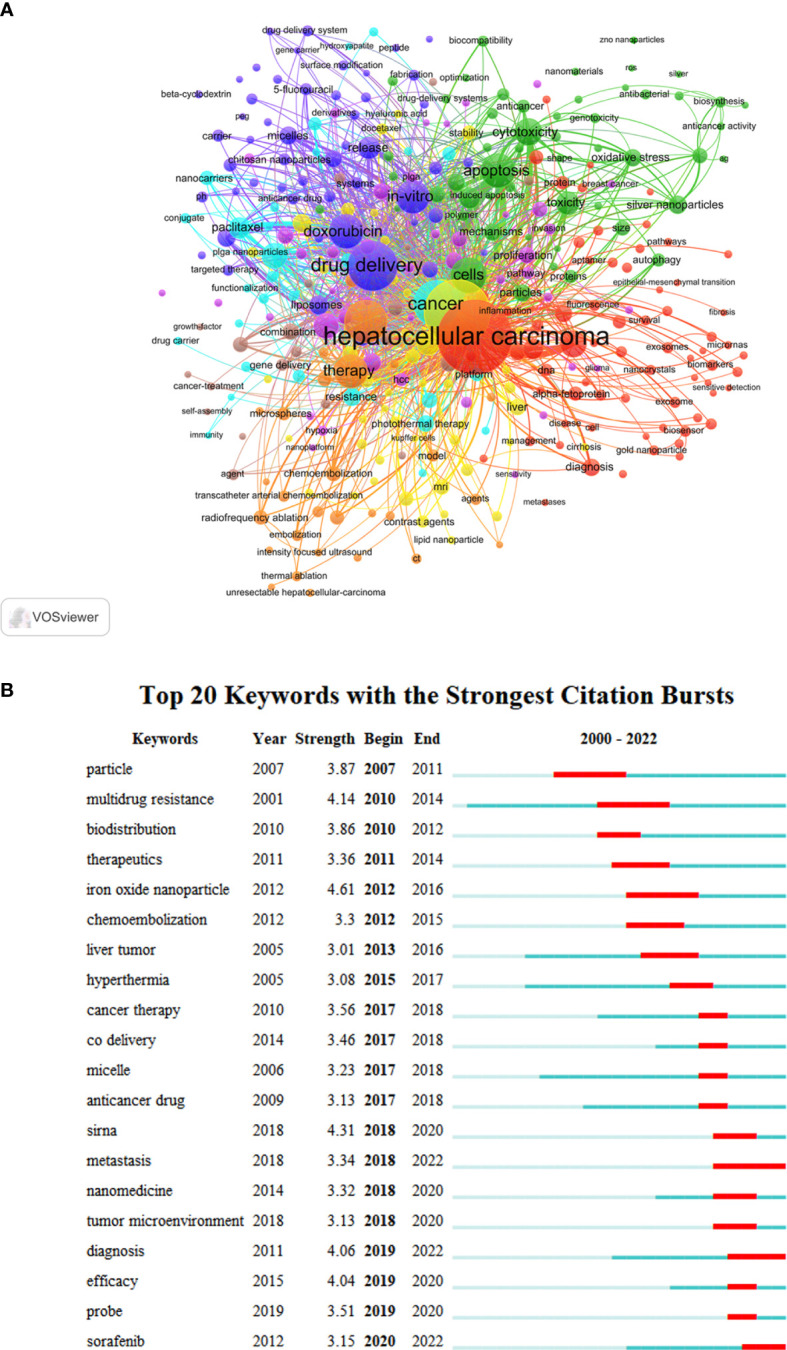
Keyword analysis of LC nanotechnology. **(A)** Keyword network graph by VOSviewer. **(B)** The top 20 keywords with the strongest citation burst.

To intuitively display these co-occurrences of high-frequency keywords (both author keywords and keywords plus), the VOSviewer software was employed. **Table 7** shows the former 20 high-frequency keywords, with “hepatocellular carcinoma” (455 times) and “nanoparticle” (370 times) having the highest frequency, followed by “drug delivery” (211 times), “cancer” (183 times) and “delivery” (163 times) (**Figure 8A**). Eight clusters were created from all the keywords (minimum of 5 occurrences per keyword), with the various colors denoting distinct research directions and objectives. The largest cluster, Cluster1 (Red), included 72 elements. It was followed by Clusters 2 (Green), 3 (Blue), 4 (Yellow), 5 (Purple), 6 (Light Blue), Cluster 7 (Orange), and Cluster 8 (brown). We utilized CiteSpace to identify the top 20 terms with the strongest citation bursts to acquire useful insights into research hotspots in this sector (**Figure 8B**). The red line plots the periods for each burst keyword, while the green line shows the period from 2000 to 2022. The most powerful explosion occurred in 2012 with iron oxide nanoparticles (burst strength 4.61) and lasted for five years. After 2018 the outbreak of citations for keywords such as “metastasis”, “diagnosis”, and “sorafenib” continued to appear, suggesting that these areas may become new research hotspots.

**Table 7:** Top 20 keywords related to the field of nanotechnology to LC.

Rank	Keyword	Occurrence	Rank	Keyword	Occurrence
1	hepatocellular carcinoma	455	11	cytotoxicity	67
2	nanoparticle	370	12	expression	65
3	drug delivery	211	13	chemotherapy	59
4	cancer	183	14	sorafenib	58
5	delivery	163	15	in-vivo	52
6	cells	115	16	toxicity	49
7	doxorubicin	107	17	release	46
8	apoptosis	107	18	paclitaxel	42
9	in-vitro	102	19	nanotechnology	42
10	therapy	100	20	growth	39

## Discussion

In recent ten years, the pathogenesis, risk factors, and treatment outcomes of LC have been a hot topics. Since 2018, research hotspots include non-alcoholic fatty liver disease (NAFLD). The cause may be that NAFLD is the most common liver disease of unknown cause worldwide, without secondary causes (e.g., drugs, excessive alcohol consumption, certain genetic disorders) (28). Non-alcoholic steatohepatitis (NASH) is an inflammatory subtype of NASH that is usually clinically asymptomatic, but over time may lead to cirrhosis, end-stage liver disease, or the need for liver transplantation. Moreover, more than 20% of patients with NASH will develop cirrhosis during their lifetime (29). Therefore, the diagnosis and treatment of liver cancer poses a great challenge.

Nanomaterials have been widely used in electronics, computers, engineering, the military, and medicine. Nanomedicine has played a major role in human health, especially in cancer applications. Cancer nanotechnology focuses on improving cancer detection, diagnosis, imaging, and treatment through nanoparticles and nanostructures while reducing the toxicity associated with conventional cancer treatments (30, 31). Rapid advances in cancer nanotechnology can be seen in the delivery of drugs *in vivo*, the development of new nanomaterials, and deeper studies of the pharmacokinetics of nanoparticles (32–34).

In this study, a total of 921 relevant articles from January 2000 to November 2021 were analyzed through scientometrics for the first time. The results showed that the number of annual publications on nanomaterials in LC has shown an increasing trend over the past few years, indicating that more and more researchers are investigating the application of nanomaterials in LC and it is gradually becoming a study hotspot in the field of LC. The two nations with the highest levels of LC nanotechnology productivity are the United States and China. The most cited author is Llovet JM, followed by Bruix J. Llovet JM for his role in developing HCC guidelines (35), exploring HCC treatments (36), and immunotherapy mechanisms (37) making a great contribution. The research of Bruix J concentrated on the establishment of HCC management guidelines and specific agents for the treatment of HCC, which has important implications for influencing the direction of HCC nanotechnology research (38, 39). The top three keywords with high frequency and centrality are “metastasis”, “diagnosis”, and “sorafenib”, which will become an emerging research focus in LC nanotechnology.

Research articles are built on keywords, and by examining keywords, one may summarize the major study issues in a certain field as well as identify research trends and hotspots (40). It is well known that the use of nanotechnology in the treatment and diagnosis of cancer is a relatively recent development that holds great promise. In this study, we identified research hotspots and development trends in nanomaterials in LC based on frequently occurring keywords (“hepatocellular carcinoma”, “nanoparticle”, “drug delivery”, “cancer”, “drug delivery”), cluster analysis of keyword co-occurrence, and keywords bursts in recent years (“metastasis”, “diagnosis”, “sorafenib”). Mainly including HCC, application of nanotechnology in LC(including liver metastases) drug delivery systems and diagnostics.

### HCC

HCC is one of the leading causes of death from cancer worldwide, and multiple risk factors contribute to the complexity and refractory nature of HCC (41). HCC is fed by a dual blood supply, the hepatic artery, and portal vein, making HCC prone to progression and metastasis compared to other gastrointestinal malignancies. Surveillance and diagnosis of HCC are based on imaging and tumor markers in the blood (42). New strategies for the treatment of HCC include surgical resection, liver transplantation, arterial chemoembolization, systemic therapy, maintenance therapy, and immunotherapy (43). However, because most HCC is diagnosed clinically at advanced stages, existing diagnostic and therapeutic approaches do not adequately meet clinical needs (44). Therefore, there is a clinical need to combine the advantages of nanotechnology, such as high individual selectivity, strong active targeting ability, and integration of diagnosis and treatment. Combining nanomedicine with a range of novel therapeutic approaches can enable personalized treatment of HCC.

### Imaging and diagnostic nanotechnology for LC

Most patients with LC are diagnosed at an advanced stage, which makes the treatment of the disease extremely difficult. Therefore, it is crucial to be able to make an accurate diagnosis of early LC. Nanotechnology has gained a lot of interest in recent years for its development and use in the field of biomedicine, particularly for the imaging and diagnosis of various malignancies. It is characterized by good penetration and tumor enrichment, which allows real-time, dynamic, and visualized tumor photography. In the field of primary LC, nanoparticles are modified as highly tumor-specific imaging contrast agents and targeted therapeutic agents (44). In a CRISPR/Cas9-induced LC mouse model, nanoparticle contrast agent-based micro-CT enables longitudinal imaging of appropriate LC size (45). In allogeneic model imaging studies established in LC patients, nanoparticles using rare earth as T2-weighted imaging contrast agents were shown to be effective in enhancing the signal difference between LC tissue and normal liver tissue on magnetic resonance imaging (MRI) (46). In addition, nano molecules are synergistic and will irreversibly dissociate to fluorescence in an acidic environment, which can be detected in subjects with solid tumors such as colon cancer, breast cancer, and LC by imaging with a fluorescent camera system, but still represents a great challenge for occult lesions (47). Therefore, the use of multiple forms of nanomaterials imaging will become a hot spot for research in early-stage LC, occult lesions, and diffuse LC borders. Based on the fact that nanoparticles have magnetic, radioactive, or plasmonic properties, gives them unique advantages in diagnostics and imaging. This enables nanomedicine to offer infinite capabilities for the diagnosis of LC.

### Nanoparticle drug delivery system in liver metastasis

A significant factor in cancer-related morbidity and mortality is liver metastasis (48). Therapeutic methods that utilize nanomedicine have been shown to deliver and maintain the bio-distribution and accumulation of therapeutic agents at the desired target site with high specificity and efficiency (49). miRNAs that are particular to the liver and have a broad effect on the micro-environment of the liver, like miR-122, are important regulators of many different liver processes. miR-122 delivery has been associated with the downregulation of key genes involved in metastatic and cancer inflammatory pathways, including several inflammatory factors, matrix metalloproteinases, and other extracellular matrix-degrading enzymes. Galactose-based miR-122 targeting lipid calcium phosphate (Gal-LCP) nanoparticles were linked to elevated CD8+/CD4+ T cell ratios and decreased immunosuppressive cell infiltration, making the liver more receptive to antitumor immune responses, which may have successfully stopped colorectal cancer liver metastases and extended survival (50, 51).

In addition, one of the most significant phases in the development of liver metastasis is the phenotypic alteration of hepatic sinusoidal endothelial cells. The expression of miRNA-20a is repressed during colorectal liver metastasis. A delivery system of chondroitin sulfate-sorbitol ester nanoparticles coupled to miR-20a restores miR-20a to normal levels in liver sinusoidal endothelial cells and induces down-regulation of its target protein expression, thus achieving a significant reduction in tumor infiltration (52). In addition, it was found that in a dimethylnitrosamine-induced HCC model, a nano-drug (NpRg3) coupled with ginsenoside Rg3 could restore the imbalance of intestinal flora caused by HCC during treatment, thus effectively inhibiting the metastasis of HCC to the lung and prolonging the survival of HCC mice (53).

Since the liver is the largest gland in the body, it is subject to both hepatic artery and portal vein blood supply, and therefore, possesses unusually rich blood flow characteristics, making the liver a common site of cancer metastasis. However, not much research has been done on nanotechnology in liver metastasis, which will become a hot spot for future research by scholars.

### Drug delivery by nanoparticles in LC

The main clinical issue that contributes to the failure of cancer treatments is multi-drug resistance, which is characterized by resistance to numerous anticancer medicines with different structural relationships (54). Chemotherapy remains the most commonly used treatment for LC, and long-term chemotherapy exhibits resistance, making it impossible to achieve satisfactory efficacy with conventional chemotherapeutic agents. Nanoparticle drug delivery is an emerging tool that offers great advantages in overcoming multi-drug resistance in the treatment of LC. A novel drug delivery system based on nanotechnology and LC micro-environment is being developed, which shows better *in vitro* anti-proliferative ability against human LC cells and high anti-tumor efficiency in nude mice, and is a promising nanomedicine for the treatment of LC (55). In the early 1990s, nanoparticle drug delivery systems began to be used in clinical practice. Doxil^®^ is the first nanomedicine that the FDA has approved, marking a significant development in the field of nanomedicine (56). The new generation of nanoparticles has entered clinical trials and received approval for several purposes over the past few decades.

We used CiteSpace to identify burst keywords to understand the change of research trends in the field over time and the hotspots of research, as shown in **Figure 8B**. The emergence of burst keywords during the past two decades demonstrates the continuous progress in LC nanotechnology research in the field. Based on this, we found that the keyword “sorafenib” was identified as the only outbreak keyword in 2020. Sorafenib, a kinase inhibitor, can be used as a novel multi-targeted oral drug for the treatment of tumors. It selectively targets the receptors of certain proteins, thus inhibiting the proliferation and angiogenesis of tumor cells and inducing apoptosis. This can also improve the survival rate of patients with advanced LC. Clinically used mainly for the treatment of inoperable or distant metastatic hepatocellular cancer (57), sorafenib is believed to act as a molecular switch in the tumor growth process. According to these signs, the FDA in the United States has given sorafenib “fast track” approval status. However, due to its poor solubility, rapid metabolism, and low bioavailability, the clinical application of sorafenib has been greatly limited (58). Therefore, improving the therapeutic effect of sorafenib will be a hot topic in future research. For example, polymer nanoparticles, lipid nanoparticles, silica nanoparticles, metal nanoparticles, and other new technologies are used to improve the targeting efficiency of sorafenib in LC and promote the development of nanomedicine in the treatment of LC. This may explain why sorafenib has become a keyword with the strongest citation burst in recent years.

Bibliometric research is a methodology for describing the evolution of structural relationships in scientific knowledge, which illustrates the many implicitly complex relationships between knowledge clusters (59). Thus, through the understanding of these complex knowledge connections, the researchers can get the hot spots and trends in a particular domain. Our bibliometrics study suggests that the use of nanotechnology to improve the efficacy of nanodelivery systems such as sorafenib and the precise diagnosis of LC (including liver metastases) may be a hot topic and research direction in this field in the coming years.

## Limitations

On the one hand, publications from other sources will be ignored as the data were obtained from the WOS database only. On the other hand, the relevant literature for this study is restricted to the period from 2000 to November 2022, while the WOS database is under constant updating. In addition, the type of literature is limited to articles and only publications in English were selected, thus leading to some bias. However, it does not affect the general trend of this study.

## Conclusion

This study provides the first comprehensive analysis of the work on nanotechnology in LC applications over the past two decades using scientific bibliometric methods and identifies research trends and hot spots in this field. Overall, the number of publications on nanotechnology in LC has been on the rise in recent years. The keyword analysis shows that the application of nanotechnology in diagnostic and drug delivery systems for LC (including liver metastases) is the current research hotspot in this field. The combined application of multiple nanotechnologies in early screening, precise diagnosis, and personalized treatment of LC (including liver metastasis) is a future issue of interest. We hope that future research will bring new hope to LC patients.

## Data availability statement

The original contributions presented in the study are included in the article. Further inquiries can be directed to the corresponding author.

## Author contributions

ZC and XJ designed the study. XJ, HL, JS, and MZ collected the data. XJ, JZ, MZ, and ZC analyzed the data and drafted the manuscript. JZ, HL, and ZC revised and approved the final version of the manuscript. All authors contributed to the article and approved the submitted version.
