# Supplementary material for: Research trends and hot spots in global nanotechnology applications in liver cancer: a bibliometric and visual analysis (2000-2022)
Source: Front Oncol. 2023 Jul 28;13:1192597. doi: 10.3389/fonc.2023.1192597 (PMC10472833; doi:10.3389/fonc.2023.1192597)
Supplement: Supplementary file 1 [file DataSheet_1.pdf]

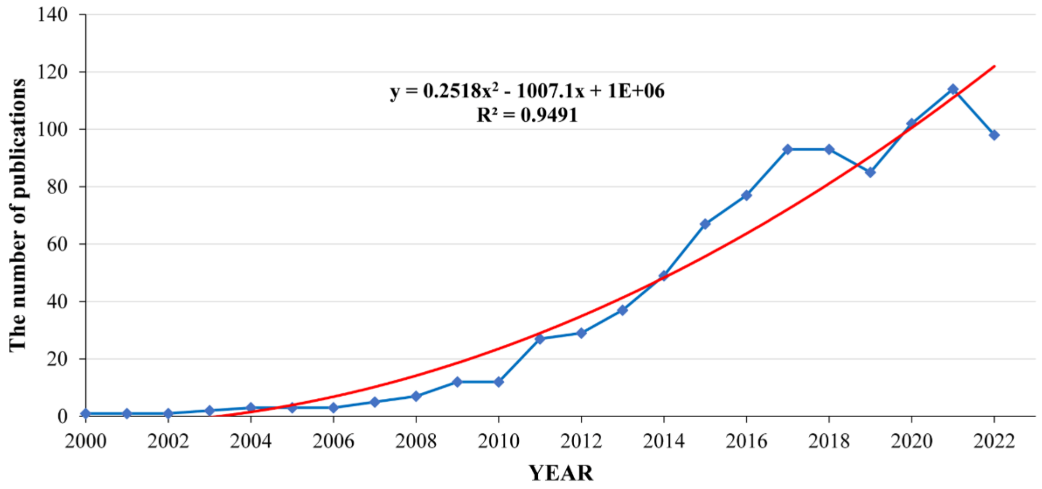


**SUPPLEMENTAL FIGURE 1**

Growth curve of LC nanotechnology research publications from January 2000 to November 2022.
